# CARING FOR OLDER INDIGENOUS PEOPLE WITH DEMENTIA: ATTENDING TO THE PERSONALITY

**DOI:** 10.1093/geroni/igz038.1621

**Published:** 2019-11-08

**Authors:** Kathleen R Mason, Tess H Moeke-Maxwell, Merryn Gott

**Affiliations:** University of Auckland, Auckland, New Zealand

## Abstract

Māori, the indigenous people of New Zealand, are living longer and dying older. The prevalence of conditions associated with older people, such as Dementia are expected to increase amongst the Māori population. Pae Herenga, a qualitative research project investigating traditional Māori end-of-life care customs, identified an indigenous narrative of Dementia care, as carried out by their families. Sixty participants took part in face-to-face interviews to systematically record the traditional care customs employed by Māori families. Of these families, five experienced caring for someone with dementia. A traditional Māori family values approach based on biological connections, relationships, empathy, love, patience and inclusiveness aimed to care for the individual with Dementia as an important member of the family, and sought to maintain as much of the person’s autonomy as possible, for as long as possible. Sharing care roles between family members and maintaining connections to Māori communities helped to prevent isolation of the person with Dementia and their family members caring for them. Involvement in family and community activities, and attending to the individual’s personality and their spiritual needs were just as important as tending to their physical care needs. These findings emphasize the importance of a holistic approach to caring for indigenous people with Dementia.

